# Pain Coping Strategies Among Adult Patients with Degenerative Disc Disease

**DOI:** 10.3390/jcm15208012

**Published:** 2026-10-16

**Authors:** Weronika Rejner, Aleksandra Bala, Agnieszka Olejnik, Antonina Gottman-Narożna, Przemysław Kunert

**Affiliations:** 1 Faculty of Psychology, University of Warsaw, Banacha 2d, 02-097 Warsaw, Poland; 2 Department of Neurosurgery, Medical University of Warsaw, Banacha 1a, 02-097 Warsaw, Poland

**Keywords:** chronic pain, degenerative disc disease, pain catastrophizing, pain coping strategies

## Abstract

**Background/Objectives**: This cross-sectional observational study aimed to investigate how individuals with lumbar degenerative disc disease (DDD) cope with pain and its association with their affective state, especially in relation to the symptoms of anxiety and depression. The study also examined whether pain intensity and duration are associated with these affective symptoms and the coping strategies used. **Methods**: The study included 210 patients with chronic pain due to DDD, one day before scheduled surgery. Patients were assessed using the Hospital Anxiety and Depression Scale or Beck Depression Inventory-II with Beck Anxiety Inventory (depending on the research project), the Visual Analog Scale for pain, and the Polish adaptation of the Coping Strategies Questionnaire. **Results**: Among participants assessed with the BDI-II, 42.7% exceeded the threshold for at least mild depressive symptoms, compared with 32.3% assessed with the HADS-D. For anxiety symptoms, 73.5% of participants assessed with the BAI and 53.8% assessed with the HADS-A exceeded the threshold for at least mild symptoms. Depressive symptoms were associated with greater average pain intensity and a longer duration of pain, whereas anxiety was positively correlated with all pain measures. The most frequently reported coping strategies were coping self-statements, praying or hoping, and increasing activity levels. Catastrophizing was associated with affective symptoms and pain intensity, whereas coping self-statements were associated with lower levels of anxiety and depressive symptoms. **Conclusions**: Patients with DDD commonly experience significant emotional distress. The findings suggest that catastrophizing is strongly associated with poorer affective state and higher pain intensity, whereas coping self-statements are associated with lower levels of anxiety and depressive symptoms.

## 1. Introduction

### 1.1. Chronic Pain

Pain is an unpleasant, subjective experience influenced by biological, psychological, and social factors. Chronic pain is defined as pain that persists or recurs for more than three months [1] and is often associated with depression, anxiety, sleep disturbances, and cognitive impairment [2]. The Global Burden of Disease (GBD) study has identified low back pain as the leading cause of disability in most countries, and epidemiological studies have shown that the number of chronic low back pain cases increased by 60.4% from 1990 to 2020. Additionally, due to population changes, the number of cases is projected to increase by 36.4% by 2050 [3]. In Poland in 2022, over a million people were on sick leave due to degenerative spine diseases, and more than 25,000 were hospitalized (data from the Polish Social Insurance Institution, 2022). Understanding the factors associated with the persistence and severity of chronic pain may contribute to more effective management and prevention strategies [4].

### 1.2. Pain Coping Strategies

To eliminate or reduce the discomfort associated with pain and its consequences, people undertake various coping actions. Lazarus (1984) defined coping as a person’s behavioral, cognitive, and emotional efforts to manage specific demands (external or internal) that are appraised as exceeding their resources [5]. In line with this concept, Lazarus and Folkman (1984) described two functions of coping: an instrumental function, aimed at solving the problem, and an emotional one, focused on regulating and controlling emotional reactions [5]. According to the stress-coping concept of Endler and Parker (1990), the coping actions (strategies) a person undertakes in a stressful situation result from an interaction between the characteristics of the situation and the individual’s characteristic coping style [5]. The latter is determined by personality and remains relatively stable regardless of the situational context [5,6]. The classification developed in 1990 by Endler and Parker supplements Lazarus and Folkman’s concept by adding an avoidance style [6].

### 1.3. Adaptive and Maladaptive Strategies

In the field of research on coping with pain, strategies are often divided based on their role in adapting to chronic pain. Adaptation to chronic pain is defined as a person’s psychological well-being, ability to perform daily physical activities, and ability to maintain social roles [7,8]. Strategies considered beneficial for adaptation are active and task-focused. In contrast, some emotion-focused and avoidance strategies, particularly their passive forms, have been described as maladaptive because their goal is to cope with the emotions related to the situation rather than changing the situation itself [9]. The literature indicates that there is a link between active coping strategies and lower pain intensity [10,11], lower levels of disability [12], and depression [10,13,14]. Maladaptive strategies have been associated with pain chronicity, reduced quality of life, increased disability, and depressive symptoms [15,16]. For individuals with chronic low back pain (CLBP), active coping strategies have been linked with decreased depression, higher levels of physical activity, and less disability. Passive coping strategies, however, are associated with higher pain intensity, depression, disability, and helplessness. A prospective study found that patients with chronic low back pain who predominantly used passive coping strategies were more likely to report poorer health-related outcomes [17]. Pain catastrophizing is a key component of the Fear-Avoidance Model (FAM) and the primary risk factor for the development of chronic pain [18].

Pain catastrophizing is defined as a tendency to ruminate on, and worry about pain, magnify its severity, or feel helpless in the face of experiencing pain [19]. Patients who exhibit this type of cognitive distortion anticipate the worst possible outcomes of a situation, which triggers a fear of pain and disability and consequently leads to activity avoidance. In the long term, such behavior has been associated with motor dysfunction, anxiety and depressive symptoms, disability [20,21,22,23], and reduced quality of life [24]. In the context of coping with chronic low back pain, praying or hoping and coping self-statements were identified as the most used strategies, while reinterpreting pain sensations was the least frequently employed [25]. Previous studies conducted in Polish populations have identified praying/hoping and catastrophizing as common coping strategies among patients with neuropathic pain [26,27].

Patients with degenerative disc disease constitute a clinically distinct population due to the presence of an identified structural spinal pathology and a defined clinical diagnosis. Although these patients frequently experience chronic low back pain, they do not represent a nonspecific chronic low back pain population. Consequently, findings derived from broader CLBP samples may not fully reflect the psychosocial functioning, emotional distress, and coping behaviors observed in patients with DDD. However, relatively few studies have specifically examined the relationships between pain coping strategies, pain characteristics, and affective symptoms in patients with DDD. A more focused investigation into the psychosocial functioning of individuals with DDD would support the creation of tailored pain management strategies and improve patient access to relevant, condition-specific information.

This study aimed to examine how individuals with lumbar DDD cope with pain and its association with their affective state, especially in relation to the symptoms of anxiety and depression. The study also examined whether pain intensity and duration are associated with these affective symptoms, as well as with the coping strategies used.

## 2. Materials and Methods

### 2.1. Study Design

This cross-sectional observational study was conducted at the Department of Neurosurgery, Medical University of Warsaw, between April 2024 and April 2026. Ethical approval for this study was obtained from the Ethics Committee for Scientific Research of the Faculty of Psychology, University of Warsaw (Approval No. 16/01/2024/08, approved on 16 January 2024), and the study was conducted in accordance with the principles of the Declaration of Helsinki. This study combined data from two research projects conducted in the same clinical setting, using identical inclusion and exclusion criteria, consecutive recruitment, and the same study protocol. Project 1 included 93 participants and assessed anxiety and depressive symptoms using the Hospital Anxiety and Depression Scale (HADS). Project 2 included 117 participants and used the Beck Depression Inventory-II (BDI-II) and the Beck Anxiety Inventory (BAI). Both projects were conducted under the same ethics approval, and the pooled analyses were performed using data collected within this approved research framework.

### 2.2. Participants

The study included 210 patients diagnosed with lumbar degenerative disc disease (DDD) hospitalized for planned surgery. Exclusion criteria included the presence of neurological disorders unrelated to DDD and a pain duration of less than three months. Only patients who remained scheduled for surgical treatment at the time of recruitment were considered eligible for participation. Patients whose surgery had been canceled due to symptom remission or clinical improvement were not approached for recruitment. Of these patients, 115 were women (54.8%) and 95 were men (45.2%). The mean age of the participants was 48 years, ranging from 20 to 85 years. The baseline demographic characteristics of the study population are presented in Table 1. The participant recruitment process and reasons for exclusion are presented in Figure 1. The present study focused exclusively on preoperative psychological functioning and pain coping strategies; the surgical procedure itself, and its outcome, were outside the scope of this study and were accordingly not recorded.

### 2.3. Procedure

All participants were informed of the purpose of the study and provided written informed consent. Participation was voluntary, and individuals could withdraw at any time. Every patient who agreed to participate completed a sociodemographic and medical data form and a set of psychological questionnaires. The assessment was conducted one day before the scheduled surgical procedure. To ensure privacy, patients were instructed to complete the materials independently, without the presence of a researcher, family members, or any other individual. All data were anonymized before analysis and were accessible only to the research team.

### 2.4. Clinical Measurements

#### 2.4.1. Pain Intensity

Pain intensity was assessed using two visual analogue scales (VASs). Each VAS comprised a 10 cm horizontal line, anchored by the descriptors “no pain” at 0 and “unbearable pain” at 10, and participants were instructed to mark the line according to the intensity of their most severe pain, regardless of whether it was back pain or radicular pain. The first scale measured current pain intensity, while the second assessed the average pain intensity over the last two weeks. Scores were categorized as follows: 0 = no pain; 1–3 = mild pain; 4–6 = moderate pain; and 7–10 = severe pain [28].

#### 2.4.2. Coping Strategies

Pain coping strategies were assessed using the Polish adaptation of the Coping Strategies Questionnaire (CSQ) [29,30]. The questionnaire consists of 42 statements describing various ways of coping with pain. It assesses the following seven specific coping strategies: diverting attention, reinterpreting pain sensations, catastrophizing, ignoring pain sensations, using coping self-statements, praying or hoping, and increasing activity level. These seven strategies are assessed using six statements for each, rated on a seven-point Likert scale, with the cumulative score for each strategy ranging from 0 to 36, where higher scores indicate greater use (endorsement) of the respective coping strategy. The questionnaire also includes two additional questions assessing the patient’s self-perceived ability to control and decrease their pain.

#### 2.4.3. Psychological Distress: Anxiety/Depression

Symptoms of depression were assessed using the Polish adaptation [31] of the Beck Depression Inventory-II (BDI-II). The inventory consists of 21 self-report items, each rated on a four-point Likert scale (0–3). The scores are added to produce a total score, with higher values indicating more severe depressive symptoms. Scores were categorized using the following recommended cutoffs: 0–13 (minimal depressive symptoms); 14–19 (mild); 20–28 (moderate); and 29–63 (severe depressive symptoms).

Anxiety symptoms were measured with the Beck Anxiety Inventory (BAI; Beck et al., 1988) [32], which consists of 21 self-report items, each rated on a four-point Likert scale (0–3). Scores were categorized using the following recommended cutoffs: 0–7 (minimal anxiety symptoms), 8–15 (mild), 16–25 (moderate), 26–63 (severe anxiety symptoms).

The severity of anxiety and depression in the hospitalized patient group was assessed using the Polish version [33] of the Hospital Anxiety and Depression Scale (HADS) [34]. The questionnaire consists of 14 statements, from which scales of depression and anxiety have been extracted. Scores of 0–7 indicate no symptoms, scores of 8–10 indicate mild symptoms, and scores of 11–21 indicate moderate to severe symptoms.

### 2.5. Statistical Analysis

Statistical analyses were performed using the Statistical Product and Service Solutions. (SPSS) version 29.0.2 software (IBM Corp., Armonk, NY, USA) for macOS. Given the deviation of the analysed data from a normal distribution, nonparametric tests were used. Spearman’s rho correlation coefficient (ρ) was used to examine the strength of the correlation between variables. The Mann–Whitney U test was used to assess differences between two independent groups in the intensity of a given dependent variable. To provide insight into the practical importance of the results, effect sizes were calculated to quantify the strength of the relationships between variables. Data on depression and anxiety were collected using two sets of tools: the Beck Inventories (BDI-II and BAI) and the Hospital Anxiety and Depression Scale (HADS). This approach was implemented because the data came from two research projects that differed only in the questionnaires used to assess emotional state. To harmonize these different data sources for a unified analysis, all raw scores from the BDI-II, BAI, and the corresponding HADS subscales were converted to Z-scores. This standardization process gives single, continuous variables for depression and anxiety that are suitable for statistical analysis across all participants. Primary multivariable linear regression models were adjusted for age, sex, education, pain duration, current pain intensity, and average pain intensity. Additional analyses included project membership as a covariate. The associations between catastrophizing, affective symptoms, and pain-related variables were treated as the primary analyses of this study. All remaining analyses were exploratory in nature, and no formal correction for multiple comparisons was applied; findings from these analyses should therefore be interpreted with caution.

## 3. Results

Mean levels of pain intensity declared by patients are presented in Table 2, including current pain, average pain over the past two weeks, and pain duration (in months). The median duration of pain was 18.5 months (IQR: 5.0–69.8 months).

Table 3 presents the correlations between age and three pain-related variables (current pain, average pain, and pain duration). Positive correlations were observed between age and both current pain intensity and pain duration. No significant correlation was found between age and average pain intensity.

Spearman’s rank correlation analysis was performed to examine the relationships between pain-related variables and symptoms of depression and anxiety. As shown in Table 4, depressive symptoms were weakly positively correlated with average pain intensity over the previous two weeks and pain duration. In contrast, no significant correlation was observed with current pain intensity. Anxiety symptoms showed weak positive correlations with all three pain-related variables: current pain intensity, average pain intensity, and pain duration.

The strategies most frequently identified as highest-scoring were coping self-statements, praying or hoping, and increasing activity level. Less commonly reported strategies included catastrophizing and diverting attention. Ignoring pain sensation and reinterpreting pain sensation were the least frequently reported strategies. Because some participants obtained identical highest scores on more than one coping strategy, they contributed to more than one category. Consequently, the total number of classifications exceeded the number of participants (231 vs. 210), and the percentages reported in Table 5 are based on the total number of classifications rather than the number of participants.

The Mann–Whitney U test revealed significant gender differences in the use of two strategies (Table 6). Women scored higher than men on catastrophizing, with a small-to-medium effect size. Women also reported greater use of praying or hoping than men, although the effect size was small.

Correlation analyses were performed to examine the relationships between demographic variables and pain coping strategies. Age was weakly negatively correlated with years of education (ρ = −0.222, *p* = 0.002). Years of education were weakly negatively correlated with the use of praying/hoping (ρ = −0.172, *p* = 0.013) and weakly positively correlated with self-reported ability to decrease pain (ρ = 0.218, *p* = 0.002). In addition, a weak positive correlation was observed between age and the use of praying/hoping (ρ = 0.147, *p* = 0.034).

Next, participants were classified according to the validated cutoff values for the instrument used in their respective project. For the Beck Depression Inventory-II (BDI-II), a score of ≥14 indicated at least mild depressive symptoms. For the Beck Anxiety Inventory (BAI), a score of ≥8 indicated at least mild anxiety symptoms. For both Hospital Anxiety and Depression Scale (HADS) subscales, a score of ≥8 was used as the validated cutoff for at least mild anxiety or depressive symptoms. Instrument-specific prevalence estimates, together with 95% confidence intervals, are presented in Table 7.

Spearman’s rho correlations were calculated to examine the relationship between anxiety and depression symptom scores and the scores obtained for specific coping strategies (Table 8). Catastrophizing was positively correlated with anxiety and depression symptoms, whereas coping self-statements were negatively correlated with both outcomes. Increasing activity level was negatively correlated with depressive symptoms. Greater perceived ability to control and decrease pain was negatively correlated with both anxiety and depression symptoms.

Table 9 and Table 10 present the results of Mann–Whitney U tests comparing pain coping strategies between participants who scored above and below the established cutoff values for anxiety and depressive symptoms. Participants from Project 1 were classified using the HADS cutoff (≥8 for both the anxiety and depression subscales), indicating at least mild symptoms. Participants from Project 2 were classified using the validated BDI-II (≥14) and BAI (≥8) cutoff scores, indicating at least mild depressive and anxiety symptoms, respectively.

The Mann–Whitney U test revealed statistically significant differences between participants scoring above and below the established cutoff values for anxiety and depressive symptoms in several pain coping strategies (Table 9 and Table 10). Higher catastrophizing scores and lower coping self-statements scores were observed among participants exceeding the cutoff values. Lower perceived ability to control and decrease pain were also observed in these participants. Additionally, participants exceeding the anxiety cutoff demonstrated lower levels of increasing activity. No significant differences were observed for the remaining coping strategies.

To compare groups according to pain severity, patients were divided into two groups: non-severe (pain score < 7) and severe (pain score ≥ 7). This grouping was applied to both the average pain and current pain variables. For average pain, 88 of 210 patients were classified as having severe pain, whereas for current pain, 43 of 210 patients were classified as having severe pain. Mann–Whitney U tests were used to compare the non-severe and severe pain groups. For average pain, statistically significant differences were observed in catastrophizing (*p* = 0.036) and perceived ability to decrease pain (*p* = 0.028). Higher catastrophizing scores were observed in the severe pain group, whereas participants in the non-severe pain group reported a greater perceived ability to decrease their pain. No statistically significant differences were observed between the non-severe and severe groups based on current pain intensity.

A Spearman’s rho correlation analysis was conducted to examine the relationship between pain characteristics (intensity and duration) and various coping strategies. The results revealed a significant, positive correlation between average pain intensity and catastrophizing (ρ = 0.211, *p* = 0.002). Furthermore, a significant positive correlation was found between pain duration and reinterpreting pain sensations (ρ = 0.171, *p* = 0.016). A significant negative correlation was observed between average pain intensity and the ability to reduce pain (ρ = −0.153, *p* = 0.027). No other coping strategies showed a statistically significant correlation with pain characteristics.

To examine whether the associations between catastrophizing and affective symptoms were independent of demographic and pain-related variables, two multivariable linear regression analyses were performed. After adjustment for age, sex, education, pain duration, current pain intensity, and average pain intensity, catastrophizing remained independently associated with depressive symptoms (β = 0.639, *p* < 0.001) and anxiety symptoms (β = 0.616, *p* < 0.001). The models accounted for 48.8% and 48.9% of the variance in depressive and anxiety symptoms, respectively (R^2^ = 0.488 and R^2^ = 0.489).

Additional regression analyses including project membership as a covariate showed that catastrophizing remained significantly associated with depressive symptoms (β = 0.602, *p* < 0.001) and anxiety symptoms (β = 0.623, *p* < 0.001). Project membership was not significantly associated with depressive symptoms (β = 0.023, *p* = 0.689) or anxiety symptoms (β =0.009, *p* = 0.870). These findings suggest that the observed associations were not explained by project membership. Prevalence estimates, which depend directly on instrument-specific cutoff values, are reported separately for each instrument in Table 7.

## 4. Discussion

Chronic pain is a complex health issue affecting millions of people worldwide, severely impairing daily functioning and lowering quality of life [35]. Low back pain, often linked to disc herniation, is one of the most frequently reported complaints in clinical practice [36], and patients with a chronic form of low back pain caused by disc herniation face physical limitations and persistent discomfort, which can strain their psychological well-being [37]. The present study found that the prevalence of elevated depressive symptoms ranged from 32.3% to 42.7%, depending on the assessment instrument used. The prevalence estimated using the BDI-II (42.7%) was comparable to data from the National Spine Network (NSN) database, which reported that 38.4% of patients with disc herniation screened positive for depression [38]. The prevalence of elevated anxiety symptoms ranged from 53.8% to 73.5%, depending on the assessment instrument used. Moreover, we found that depressive symptoms were associated with greater pain intensity over the last two weeks and a longer duration of pain sensations. In contrast, anxiety was positively correlated with all pain measures, including a higher level of current pain. These findings align with previous research indicating that patients with disc herniation frequently present symptoms of depression and anxiety [39,40,41].

Our study showed that older individuals tend to report higher levels of current pain and longer durations of pain. Research on age-related pain sensitivity has yielded contradictory findings, with some studies reporting decreased sensitivity [42] and others reporting lower pain thresholds with age [43]. In medical research, it is assumed that aging is associated with progressive muscle atrophy and functional decline in lower limbs [44]. Previous research has shown that older individuals had significantly lower net relative functional muscle volume than younger ones. Additionally, reductions in net lower-limb muscle volume were positively correlated with pain intensity (VAS scores), age, and duration of pain [45]. Since our study only provides insight into the subjective intensity of pain, it does not allow conclusions regarding the mechanisms underlying the higher pain reports observed among older participants. However, factors such as muscle volume and pain sensitivity may provide a potential explanation for our results.

The results of our study showed that the most frequently reported coping strategies were self-statements, praying or hoping, and increasing activity levels. Previous studies have also identified praying or hoping as a commonly used coping strategy among individuals with chronic pain [26,27]. As participants were awaiting surgical treatment, the “praying or hoping” category may reflect not only religious coping but also hope for symptom relief and successful treatment outcomes. In our study, however, this coping strategy was not associated with pain intensity or symptoms of anxiety and depression, which is consistent with the mixed findings reported in previous research [46,47,48].

Increasing activity levels was also among the most frequently reported strategies, but as with praying/hoping, there was no association with pain intensity or symptoms of anxiety and depression. Previous studies have distinguished three activity patterns among individuals with chronic pain: task-contingent persistence, pain-contingent persistence, and excessive persistence [49]. They found that only the excessive form is associated with greater depression and disability. Excessive persistence is defined as persisting with activities despite harmful consequences and ignoring physical limits or warning signs. An increased activity level in our sample may not reflect excessive persistence, and patients may adjust their activity in a nonharmful, balanced way.

Of all the strategies that were considered in this study, catastrophizing and coping self-statements were found to be most closely related to affective state and pain intensity. Coping self-statements, the most frequently reported strategy, were associated with lower levels of anxiety and depressive symptoms. This finding is consistent with previous studies highlighting the importance of self-efficacy and adaptive coping in chronic pain populations [50,51]. However, coping self-statements were not associated with pain intensity or pain duration. In contrast, lower average pain intensity was associated with a greater perceived ability to reduce pain. The ability to reduce or control pain seems to be a different concept from coping self-statements. Coping self-statements may reinforce beliefs about effectiveness, which can be beneficial for psychological well-being but not for reducing pain intensity. The situation is different in the case of catastrophizing, which is positively associated with symptoms of anxiety and depression, and participants presenting clinically significant symptoms of anxiety or depression reported higher levels of catastrophizing. These findings are consistent with previous research conducted among patients with chronic low back pain and disc herniation [52,53,54]. Furthermore, higher levels of catastrophizing were associated with greater average pain intensity. Previous research has also shown that the relationship between pain catastrophizing and physical health varies according to pain intensity, whereas its association with psychological well-being remains relatively stable [55]. Consistent with these findings, higher levels of catastrophizing in our sample were associated with greater pain intensity. However, due to the study’s cross-sectional design, no conclusions can be drawn about the direction or causal nature of this relationship.

We noted some limitations arising from the nature of the clinical group and the study context. Participants were assessed during hospitalization while awaiting surgical treatment. This period may have been associated with specific emotional responses, including both increased distress and heightened hope and positive expectations regarding the outcomes of planned surgical intervention. To control for potential surgery-related bias in the anxiety or depression levels in questionnaires, additional items assessing the patient’s emotional response to the upcoming surgery should be considered in future research. Anxiety and depressive symptoms were assessed using different instruments across the two projects. Although score standardization enabled pooled analyses, it does not establish psychometric equivalence between the measures or their clinical cutoffs. Therefore, the pooled findings should be interpreted alongside the instrument-specific results. Detailed clinical information, including the spinal levels involved, neurological deficits, previous treatments, and the types of planned surgical procedures, was not available for the present analysis. In addition, pain intensity was assessed using VAS ratings that did not distinguish radicular pain from low back pain. Because only participant-level total and subscale scores were available in the analytical dataset, and item-level responses had not been retained, internal consistency coefficients could not be calculated for the present sample. The exploratory nature of several analyses and the lack of formal adjustment for multiple comparisons should be considered when interpreting the results.

## 5. Conclusions

The findings of the study allowed us to identify which coping strategies are most frequently used by patients with chronic pain due to degenerative disc disease. Moreover, the study highlights which coping strategies may be beneficial due to their lack of a negative association with well-being, such as coping self-statements. Another crucial finding is that catastrophizing has been identified as a significant threat for chronic pain patients. Catastrophizing is strongly associated not only with higher levels of depression and anxiety symptoms but also with reporting greater pain intensity. The observed associations suggest that catastrophizing and perceived pain control may be relevant targets for future research and clinical interventions. However, due to the cross-sectional nature of the study, no conclusions can be drawn regarding the direction of these relationships.

## Figures and Tables

**Figure 1:**
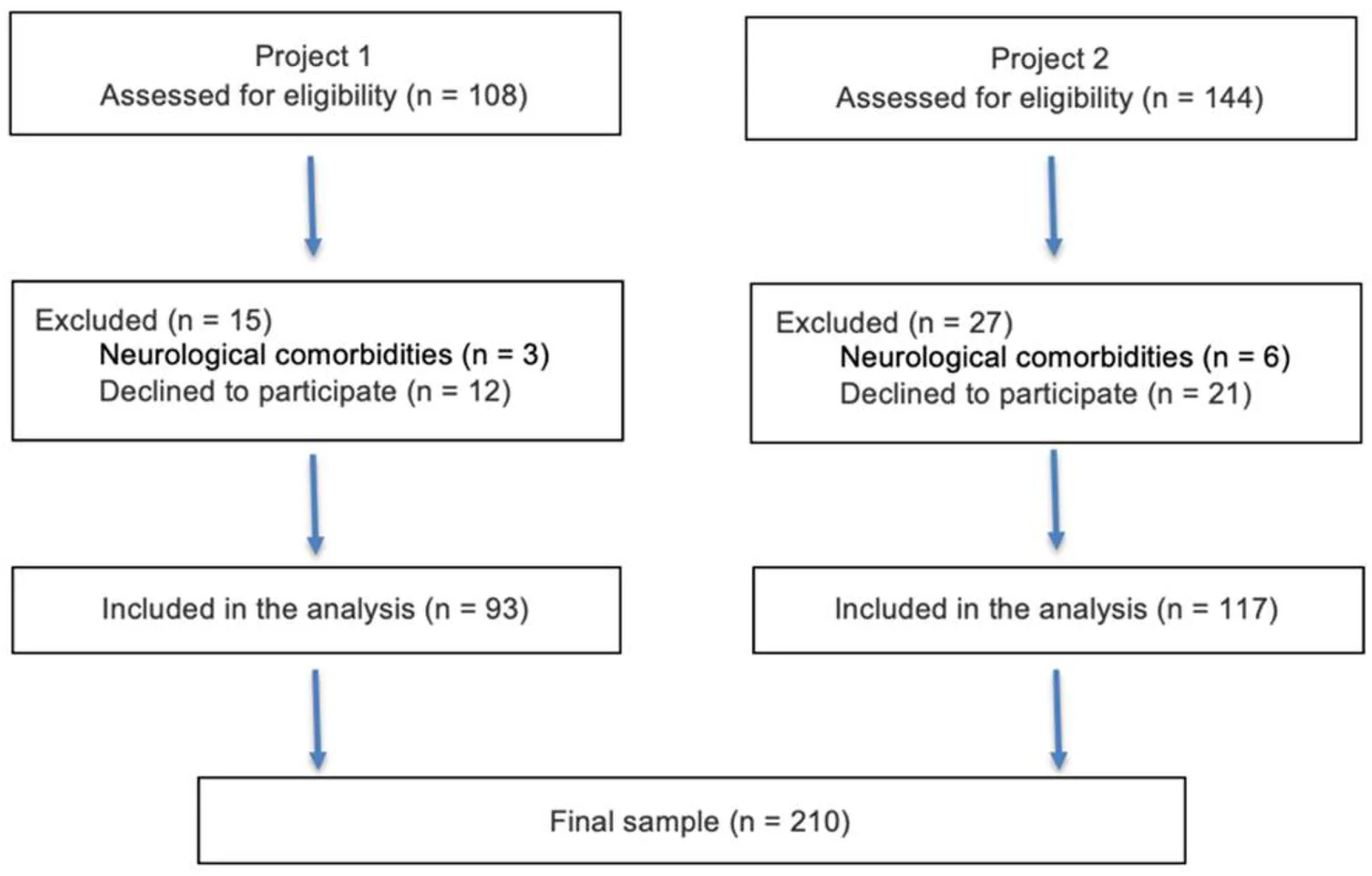
Flow diagram of participant recruitment and inclusion in the study.

**Table 1:** Demographic Data.

	Min	Max	M	SD
Age	20	85	47.93	12.88
Years of education	8	21	14.2	2.45

Min = minimum value; Max = maximum value; M = mean; SD = standard deviation.

**Table 2:** Pain Characteristics.

	M	SD
Current pain	4.49	2.55
Average pain (over 2 weeks)	5.98	2.45
Duration of pain (in months)	58.75	99.59

M = mean; SD = standard deviation.

**Table 3:** Correlation Between Different Measures of Pain and Age.

	Statistic	Current Pain	Average Pain	Duration of Pain
Age	Spearman’s rho	0.200 *	0.122	0.228 *
	*p*-value	0.004	0.078	0.001

* *p* < 0.05.

**Table 4:** Correlations Between Pain Characteristics, Depression, and Anxiety.

	Statistic	Depression	Anxiety
Current pain	Spearman’s rho	0.111	0.161 *
	*p*-value	0.112	0.019
Average pain	Spearman’s rho	0.146 *	0.229 **
	*p*-value	0.034	<0.001
Duration of pain	Spearman’s rho	0.175 *	0.170 *
	*p*-value	0.014	0.017

** *p* < 0.001; * *p* < 0.05.

**Table 5:** Distribution of the Highest-Scoring Pain Coping Strategies.

Strategy	M (SD)	Frequency (*n*)	Percentages (%)
Diverting attention	13.83 (7.73)	24	10.4
Reinterpreting pain sensation	7.04 (6.44)	2	0.9
Catastrophizing	11.46 (7.49)	25	10.8
Ignoring pain sensation	11.50 (7.36)	7	3.0
Praying or hoping	16.51 (7.43)	56	24.2
Coping-self statements	17.89 (7.45)	65	28.1
Increasing activity level	16.59 (7.22)	52	22.5
		231	

M = mean; SD = standard deviation; frequency (*n*) = number of highest-score classifications assigned to each coping strategy; percentages (%) = proportion of all classifications.

**Table 6:** Gender Differences in the Use of Strategies.

Strategy	Female (*n* = 115)		Male (*n* = 95)		Z	*p*	r
	Mdn	IQR	Mdn	IQR			
Catastrophizing	13	10	9	11	−3.32	<0.001	−0.23
Praying or hoping	17	11	15	11	−2.20	0.028	−0.15

Mdn = Median; IQR = Interquartile Range; Z = Standardized test statistic; r = Effect size.

**Table 7:** Participants Above the Established Cutoffs for Anxiety and Depressive Symptoms.

	Instrument	*n*/*N*	%	95% CI
Depression	BDI-II	50/117	42.7	34.1–51.8
	HADS-D	30/93	32.3	23.7–42.2
Anxiety	BAI	86/117	73.5	64.7–80.8
	HADS-A	50/93	53.8	43.7–63.6

95% CI = 95% confidence interval.

**Table 8:** Relationship Between Anxiety and Depression Symptom Scores and the Scores Obtained for Specific Coping Strategies.

Strategy		Depression	Anxiety
Diverting attention	Spearman’s rho	−0.072	0.002
	*p*-value	0.298	0.983
Reinterpreting pain sensation	Spearman’s rho	−0.001	0.125
	*p*-value	0.986	0.070
Catastrophizing	Spearman’s rho	0.481 **	0.532 **
	*p*-value	<0.001	<0.001
Ignoring pain sensation	Spearman’s rho	−0.126	−0.037
	*p*-value	0.069	0.594
Praying or hoping	Spearman’s rho	−0.032	0.028
	*p*-value	0.643	0.687
Coping-self statements	Spearman’s rho	−0.202 *	−0.172 *
	*p*-value	0.003	0.013
Increasing activity level	Spearman’s rho	−0.154 *	−0.106
	*p*-value	0.025	0.126
Q1: control over pain	Spearman’s rho	−0.243 **	−0.247 **
	*p*-value	<0.001	<0.001
Q2: decrease the pain	Spearman’s rho	−0.204 *	−0.203 *
	*p*-value	0.003	0.003

** *p* < 0.001; * *p* < 0.05.

**Table 9:** Differences in Pain Coping Strategies Between Patients Above and Below the Cutoff for Depressive Symptoms.

Strategy	Depression		Below Clinical Cutoff		Z	*p*	r
	(*N* = 80)		(*N* = 130)				
	Mdn	IQR	Mdn	IQR			
Diverting attention	12	11	14	12.25	−1.081	0.280	−0.08
Reinterpreting pain sensation	5	10	5	9	−0.354	0.723	−0.02
Catastrophizing	15	10	8	11	−6.425	<0.001	−0.44
Ignoring pain sensation	11	13	11	11.25	−1.171	0.242	−0.08
Praying or hoping	15	10	17	12	−0.930	0.352	−0.06
Coping-self statements	17	9	18	10	−2.380	0.017	−0.16
Increasing activity level	17	8	18	8	−1.621	0.105	−0.11
Q1: control over pain	3	1	3	1	−2.493	0.013	−0.17
Q2: decrease the pain	3	1	3	0	−3.106	0.002	−0.21

Mdn = median; IQR = interquartile range; Z = standardized test statistic; r = effect size.

**Table 10:** Differences in Pain Coping Strategies Between Patients Above and Below the Cutoff for Anxiety Symptoms.

Strategy	Anxiety		Below Clinical Cutoff		Z	*p*	r
	*N* = 136		*N* = 74				
	Mdn	IQR	Mdn	IQR			
Diverting attention	13	10	14	14	−0.578	0.563	−0.04
Reinterpreting pain sensation	6	10	4.5	10	−1.01	0.311	−0.07
Catastrophizing	13	10	7	9	−6.08	<0.001	−0.42
Ignoring pain sensation	10	10	12	13	−1.71	0.087	−0.12
Praying or hoping	16	10	16	12	−0.861	0.389	−0.06
Coping-self statements	17	9	20	9	−3.59	<0.001	−0.25
Increasing activity level	17	8	19	8	−2.31	0.021	−0.16
Q1: control over pain	3	0	3	1	−2.57	0.010	−0.18
Q2: decrease the pain	3	1	3	0	−2.27	0.023	−0.16

Mdn = median; IQR = interquartile range; Z = standardized test statistic; r = effect size.

## Data Availability

The data presented in this study are available on reasonable request from the corresponding author.

## Abbreviations

The following abbreviations are used in this manuscript:

DDD	Degenerative Disc Disease
CLBP	Chronic Low Back Pain
FAM	Fear Avoidance Model
VAS	Visual Analogue Scale
CSQ	Coping Strategies Questionnaire
BAI	Beck Anxiety Inventory
BDI-II	Beck Depression Inventory-II
HADS	Hospital Anxiety and Depression Scale

## References

[1] Nicholas M., Vlaeyen J.W., Rief W., Barke A., Aziz Q., Benoliel R., Cohen M., Evers S., Giamberardino M.A., Goebel A. (2019). The IASP classification of chronic pain for ICD-11: Chronic primary pain. Pain.

[2] Amaro-Díaz L., Montoro C.I., Fischer-Jbali L.R., Galvez-Sánchez C.M. (2022). Chronic Pain and Emotional Stroop: A Systematic Review. J. Clin. Med..

[3] Ferreira M.L., De Luca K., Haile L.M., Steinmetz J.D., Culbreth G.T., Cross M., Kopec J.A., Ferreira P.H., Blyth F.M., Buchbinder R. (2023). Global, regional, and national burden of low back pain, 1990–2020, its attributable risk factors, and projections to 2050: A systematic analysis of the Global Burden of Disease Study 2021. Lancet Rheumatol..

[4] Todd J., Pickup B., Coutts-Bain D., Duijzings M., Sharpe L. (2025). Interpretation bias and its relationship with pain: A systematic review and meta-analysis. Pain.

[5] Endler N.S., Parker J.D. (1990). Multidimensional assessment of coping: A critical evaluation. J. Pers. Soc. Psychol..

[6] Endler N.S., Parker J.D. (1994). Assessment of multidimensional coping: Task, emotion, and avoidance strategies. Psychol. Assess..

[7] Geisser M.E., Robinson M.E., Riley J.L. (1999). Pain Beliefs, Coping, and Adjustment to Chronic Pain: Let’s Focus More on the Negative. Pain. Forum.

[8] Sturgeon J.A., Zautra A.J. (2010). Resilience: A new paradigm for adaptation to chronic pain. Curr. Pain Headache Rep..

[9] Carroll L. (2020). Problem-Focused Coping. Encyclopedia of Behavioral Medicine.

[10] Higgins N.C., Bailey S.J., LaChapelle D.L., Harman K., Hadjistavropoulos T. (2015). Coping styles, pain expressiveness, and implicit theories of chronic pain. J. Psychol..

[11] Ramίrez-Maestre C., Esteve R., López A.E. (2012). The role of optimism and pessimism in chronic pain patients adjustment. Span. J. Psychol..

[12] Nicholas M.K., Blyth F.M. (2016). Are self-management strategies effective in chronic pain treatment?. Pain Manag..

[13] Kuzu D., Kim S., Kratz A.L. (2022). Active or passive pain coping: Which predicts daily physical and psychosocial functioning in people with chronic pain and spinal cord injury?. Rehabil. Psychol..

[14] Miller-Matero L.R., Chipungu K., Martinez S., Eshelman A., Eisenstein D. (2017). How do I cope with pain? Let me count the ways: Awareness of pain coping behaviors and relationships with depression and anxiety. Psychol. Health Med..

[15] Higuchi D. (2020). Adaptive and maladaptive coping strategies in older adults with chronic pain after lumbar surgery. Int. J. Rehabil. Res..

[16] Tan G., Teo I., Anderson K.O., Jensen M.P. (2011). Adaptive versus maladaptive coping and beliefs and their relation to chronic pain adjustment. Clin. J. Pain.

[17] Hall A., Pike A., Bursey K., Mortazhejri S., Patey A., Grimshaw J., Etchegary H. (2025). An exploration of patients’ perceptions and coping strategies for LBP. PLoS ONE.

[18] Vlaeyen J.W., Linton S.J. (2000). Fear-avoidance and its consequences in chronic musculoskeletal pain: A state of the art. Pain.

[19] Sullivan M.J., Martel M.O., Tripp D., Savard A., Crombez G. (2006). The relations between catastrophizing and the communication of pain experience. Pain.

[20] Alodaibi F.A., Minick K.I., Fritz J.M. (2013). Do preoperative fear avoidance model Factors predict outcomes after lumbar disc herniation surgery? A systematic review. Chiropr. Man. Therap..

[21] Bunzli S., Smith A., Schütze R., Lin I., O’Sullivan P. (2017). Making sense of low back pain and pain-related fear. J. Orthop. Sports Phys. Ther..

[22] Koechlin H., Coakley R., Schechter N., Werner C., Kossowsky J. (2018). The role of emotion regulation in chronic pain: A systematic literature review. J. Psychosom. Res..

[23] Ramίrez-Maestre C., Esteve R. (2014). The role of sex/gender in the experience of pain: Resilience, fear, and acceptance as central variables in the adjustment of men and women with chronic pain. J. Pain.

[24] Ionescu D., Iacob C.I., Brehar F.M., Avram E. (2023). The role of catastrophizing and basic psychological needs satisfaction on health-related quality of life and pain in patients with lumbar disc herniation. Front. Psychol..

[25] Rosenstiel A.K., Keefe F.J. (1983). The use of coping strategies in chronic low back pain patients: Relationship to patient characteristics and current adjustment. Pain.

[26] Kołpa M., Mężyk E. (2016). Strategies for coping with neuropathic pain and impact of pain on the functional condition of patients. Pielęgniarstwo Neurol. Neurochir..

[27] Kryszak A., Czernicki Z., Wiśniewski D. (2021). Psychological Assessment of Control and Coping with Neuropathic Pain in the Lumbar Spine. Med. Rehabil..

[28] Serlin R.C., Mendoza T.R., Nakamura Y., Edwards K.R., Cleeland C.S. (1995). When is cancer pain mild, moderate or severe? Grading pain severity by its interference with function. Pain.

[29] Juczyński Z., Ogińska-Bulik N. (2012). Narzędzia Pomiaru Stresu i Radzenia Sobie ze Stresem.

[30] Juczyński Z. (2012). Narzędzia Pomiaru w Promocji i Psychologii Zdrowia.

[31] Beck A.T., Steer R.A., Brown G.K., Stańczak J., Jaworowska A. (2019). Beck Depression Inventory–II (BDI-II).

[32] Beck A.T., Epstein N., Brown G., Steer R.A. (1988). An inventory for measuring clinical anxiety: Psychometric properties. J. Consult. Clin. Psychol..

[33] Majkowicz M., de Walden-Gałuszko K., Majkowicz M. (2000). Praktyczna ocena efektywności opieki paliatywnej—Wybrane techniki badawcze. Ocena Jakości Opieki Paliatywnej w Teorii i w Praktyce.

[34] Zigmond A.S., Snaith R.P. (1983). The hospital anxiety and depression scale. Acta Psychiatr. Scand..

[35] Cohen S.P., Vase L., Hooten W.M. (2021). Chronic pain: An update on burden, best practices, and new advances. Lancet.

[36] Wei Y., Huang H., Sun K., Gao H., Cao Z., Zhang B., Wu J., Liu Y. (2024). Analysis of preoperative and postoperative depression and anxiety in patients with lumbar disc herniation with radiculopathy treated with percutaneous transforaminal endoscopic discectomy. Front. Psychiatry.

[37] Munakomi S., Foris L.A., Varacallo M.A. (2023). Spinal Stenosis and Neurogenic Claudication.

[38] Levy H.I., Hanscom B., Boden S.D. (2002). Three-question depression screener used for lumbar disc herniations and spinal stenosis. Spine.

[39] Cai L., He Q., Lu Y., Hu Y., Chen W., Wei L., Hu Y. (2019). Comorbidity of pain and depression in a lumbar disc herniation model: Biochemical alterations and the effects of fluoxetine. Front. Neurol..

[40] Kao Y.C., Chen J.Y., Chen H.H., Liao K.W., Huang S.S. (2022). The association between depression and chronic lower back pain from disc degeneration and herniation of the lumbar spine. Int. J. Psychiatry Med..

[41] Mu W., Shang Y., Zhang C., Tang S. (2019). Analysis of the depression and anxiety status and related risk factors in patients with lumbar disc herniation. Pak. J. Med. Sci..

[42] González-Roldán A.M., Terrasa J.L., Sitges C., van der Meulen M., Anton F., Montoya P. (2020). Age-related changes in pain perception are associated with altered functional connectivity during resting state. Front. Aging Neurosci..

[43] Zhi Y., Zhang Y., Zhang Y., Zhang M., Kong Y. (2024). Age-associated changes in multimodal pain perception. Age Ageing.

[44] Valentin S., Licka T., Elliott J. (2015). Age and side-related morphometric MRI evaluation of trunk muscles in people without back pain. Man. Ther..

[45] Chen Z.N., Yao X.M., Lv Y., He B.J., Ye J.C., Shao R.X., Jiang H.W. (2018). Morphology of the lumbar multifidus muscle in lumbar disc herniation at different durations and at different ages. Exp. Ther. Med..

[46] Wachholtz A.B., Pearce M.J., Koenig H. (2007). Exploring the relationship between spirituality, coping, and pain. J. Behav. Med..

[47] Najem C., Mukhtar N.B., Ayoubi F., Van Oosterwijck J., Cagnie B., De Meulemeester K., Meeus M. (2021). Religious beliefs and attitudes in relation to pain, pain-related beliefs, function, and coping in chronic musculoskeletal pain: A systematic review. Pain Physician.

[48] Delity M.J., Wachholtz A., Webb J.R. (2025). Spirituality and Chronic Medical Illness. Handbook of Spirituality, Health, and Well-Being.

[49] Nielson W.R., Jensen M.P., Karsdorp P.A., Vlaeyen J.W. (2013). Activity pacing in chronic pain: Concepts, evidence, and future directions. Clin. J. Pain.

[50] Woby S.R., Urmston M., Watson P.J. (2007). Self-efficacy mediates the relation between pain-related fear and outcome in chronic low back pain patients. Eur. J. Pain.

[51] Jackson T., Wang Y., Wang Y., Fan H. (2014). Self-efficacy and chronic pain outcomes: A meta-analytic review. J. Pain.

[52] Ranger T.A., Cicuttini F.M., Jensen T.S., Manniche C., Heritier S., Urquhart D.M. (2020). Catastrophization, fear of movement, anxiety, and depression are associated with persistent, severe low back pain and disability. Spine J..

[53] Wertli M.M., Rasmussen-Barr E., Held U., Weiser S., Bachmann L.M., Brunner F. (2014). Fear-avoidance beliefs—A moderator of treatment efficacy in patients with low back pain: A systematic review. Spine J..

[54] Suso-Ribera C., García-Palacios A., Botella C., Ribera-Canudas M.V. (2017). Pain catastrophizing and its relationship with health outcomes: Does pain intensity matter?. Pain Res. Manag..

[55] Shin D.A., Chang M.C. (2023). Five reasons why some herniated intervertebral discs are painless. J. Pain Res..

